# Evaluating Nuclear Membrane Irregularity for the Classification of Cervical Squamous Epithelial Cells

**DOI:** 10.1371/journal.pone.0164389

**Published:** 2016-10-14

**Authors:** Jing Rui Tang, Nor Ashidi Mat Isa, Ewe Seng Ch’ng

**Affiliations:** 1 Imaging and Intelligent Systems Research Team, School of Electrical and Electronic Engineering, Universiti Sains Malaysia, Nibong Tebal, Pulau Pinang, Malaysia; 2 Advanced Medical and Dental Institute, Universiti Sains Malaysia, Bertam, Kepala Batas, Pulau Pinang, Malaysia; Federal University of Rio de Janeiro, BRAZIL

## Abstract

Pap test involves searching of morphological changes in cervical squamous epithelial cells by pathologists or cytotechnologists to identify potential cancerous cells in the cervix. Nuclear membrane irregularity is one of the morphological changes of malignancy. This paper proposes two novel techniques for the evaluation of nuclear membrane irregularity. The first technique, namely, penalty-driven smoothing analysis, introduces different penalty values for nuclear membrane contour with different degrees of irregularity. The second technique, which can be subdivided into mean- or median-type residual-based analysis, computes the number of points of nuclear membrane contour that deviates from the mean or median of the nuclear membrane contour. Performance of the proposed techniques was compared to three state-of-the-art techniques, namely, radial asymmetric, shape factor, and rim difference. Friedman and post hoc tests using Holm, Shaffer, and Bergmann procedures returned significant differences for all the three classes, i.e., negative for intraepithelial lesion or malignancy (NILM) versus low grade squamous intraepithelial lesion (LSIL), NILM versus high grade squamous intraepithelial lesion (HSIL), and LSIL versus HSIL when the span value equaled 3 was employed with linear penalty function. When span values equaled 5, 7, and 9, NILM versus LSIL and HSIL showed significant differences regardless of the penalty functions. In addition, the results of penalty-driven smoothing analysis were comparable with those of other state-of-the-art techniques. Residual-based analysis returned significant differences for the comparison among the three diagnostic classes. Findings of this study proved the significance of nuclear membrane irregularity as one of the features to differentiate the different diagnostic classes of cervical squamous epithelial cells.

## 1.0 Introduction

Papanicolaou test (Pap test) is a screening test for cervical cancer aiming to identify pre-cancerous and cancerous cells in the cervix. Named after the inventor, George Papanicolaou, Pap test has significantly reduced the mortality rate owing to cervical cancer [1]. The test involves the search of morphological alterations related to malignancy in cervical squamous epithelial cells by pathologists or cytotechnologists. The nuclei of cancerous cells often demonstrate characteristics that are observable under light microscope, such as changes in the nuclear shape, nuclear size, and chromatin distribution [2–6].

The nucleus of non-neoplastic cell is generally round, oval, or bean-shaped. Conversely, nuclear membrane irregularity, an aspect of pleomorphism in terms of variability in shapes, is commonly seen in malignant nucleus [7–16]. Irregularity may appear as nuclear grooving, nuclear molding, or nuclear convolutions [2]. Nuclear shape is controlled by the forces from nuclear envelope, nuclear matrix, and cytoskeleton [17]. Judgment of nuclear membrane irregularity is highly subjective and depends on how individual pathologists interpret and define the shape of the nucleus. Based on the observation of numerous cases, pathologists are trained to differentiate between “normal” and “abnormal” contours of the nuclear membrane. Acknowledging that recognition and discrimination between the “normal” and “abnormal” contours of the nuclear membrane may help in rapid examination of Pap smear, this study aims to propose simple yet effective methods of defining nuclear membrane irregularity and further justify the significance of nuclear membrane irregularity in differentiating the different diagnostic classes of cervical squamous epithelial cells.

This paper presents two techniques to evaluate nuclear membrane irregularity. The first technique assumes that the nuclear membrane contour of a normal cell is smoother than those of the abnormal cell. Therefore, the technique compares the nuclear membrane contour with a “smoothed” contour, which is derived from the original nuclear membrane contour. The second technique is designed based on the assumption that the nucleus of a normal cell is round in shape. The amount of deviation of the nuclear membrane contour from the mean or median value of its nuclear membrane contour will determine to which categories the cell belong (i.e., negative for intraepithelial lesion or malignancy (NILM), low grade squamous intraepithelial lesion (LSIL), or high grade squamous intraepithelial lesion (HSIL)). A total of 600 test images (200 test images from NILM, LSIL, and HSIL, respectively, as attached in S1–S3 Files) were used to evaluate the performance of the proposed techniques. Statistical analysis was performed to seek any significant differences among the three diagnostic classes. Studies have reported various methods of nuclear shape evaluation [18–22]. Performance of the proposed techniques was compared with three techniques of nuclear shape evaluation in the literature, namely, radial asymmetric (RA) [18], shape factor (SF) [18], and rim difference (RD) [23]. Briefly, a technique employing RA initially computes the centroid of the nuclear area to be used as the center to inscribe the largest circle in a nuclear area. RA is the proportion of the nuclear pixels lying outside the inscribed circle [18]. SF, which is mathematically expressed as Eq (1), is another nuclear shape metric in [18]. SF of a perfect circle is one. CYBEST4, which is one of the automated cytologic screening systems, evaluates nuclear shape based on the assumption that the length of the nuclear membrane contour is shorter for regular nucleus compared with irregular nucleus with the same area [23]. Nuclear membrane irregularity is defined in term of RD.

$$SF=\frac{Perimeter^{2}}{4\times \pi \times Area}$$ (1)

where *Perimeter* and *Area* are the perimeter and area of the nucleus, respectively, and *π* is a mathematical constant with a value of approximately 3.14159 and represents the ratio of a circle circumference to its diameter.

The next section presents the methodology of the proposed techniques. Section 3 outlines the simulation results followed by the discussions in Section 4. Finally, Section 5 concludes our work.

## 2.0 Methodology

Two techniques, namely, penalty-driven smoothing analysis and residual-based analysis, were proposed to evaluate nuclear membrane irregularity. The study involved three main stages, namely, (1) data acquisition, (2) processing of cervical squamous epithelial cell images, and (3) evaluation of nuclear membrane irregularity. All processing steps of cervical squamous epithelial cell images were performed using MATLAB version R2015a. Details of each stage are presented in the following sub-sections.

### 2.1. Acquisition of Cervical Squamous Epithelial Cell Images

The study was approved by the Human Research Ethics Committee of Universiti Sains Malaysia with the reference code USMKK/PPP/JEPeM[217.4(2.6)]. Human Research Ethics Committee of Universiti Sains Malaysia is listed under the Office for Human Research Protections (OHRP), United States Department of Health and Human Services. The Federal-wide Assurance (FWA) identification number is FWA00007718 and the Institutional Review Board (IRB) number is IRB00004494. ThinPrep slides were borrowed from Penang General Hospital and Tuanku Fauziah Hospital in Malaysia. A total of 102 slides were borrowed (namely, 37 slides from NILM, 42 slides from LSIL, and 23 slides from HSIL). The slides had been previously read and screened by at least a cytotechnologist and a pathologist and formally reported as NILM, LSIL, or HSIL. Cells from NILM, LSIL, and HSIL classes were then individually picked by a cytotechnologist and reconfirmed by a pathologist. The slides were reviewed without knowledge on the patients’ background and history; therefore, no consent was obtained from the patients. Cells were selected according to the set of criteria in the Bethesda system [24]. A total of 600 images, consisting of two hundred images for each diagnostic class, were captured from the 102 ThinPrep slides. Images were captured using an Olympus BX43F clinical microscope mounted with a video camera. Every cell image was zoomed with 100× objective with oil immersion.

### 2.2 Processing of Cervical Squamous Epithelial Cell Images

Processing of cervical squamous epithelial cell images included image enhancement and nucleus segmentation. The cervical squamous epithelial cell image that was captured from ThinPrep slide was initially cropped for the nucleus region and then converted from color to gray level image to reduce computational burden. Histogram equalization was then performed to enhance the contrast of the image.

**Fig 1:**
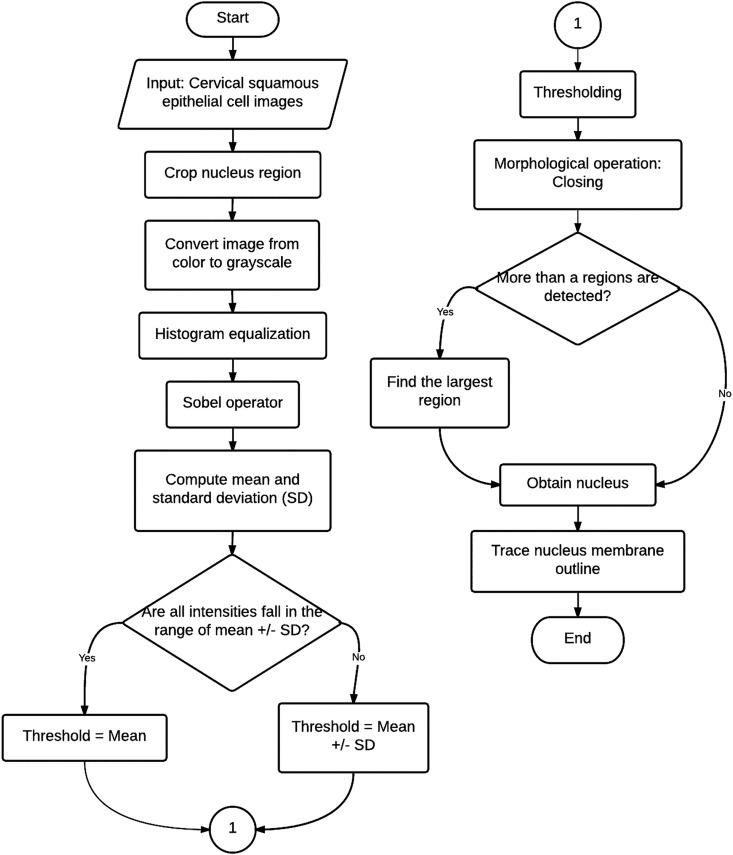
Flowchart for the processing of cervical squamous epithelial cell images.

After the image was pre-processed, gradient of the image was computed using the Sobel operator. Mean and standard deviation of the gradient image were computed. The summation and the difference between these mean and standard deviation values were computed as well. If the intensities of the entire gradient image fell in range of the computed difference and the summation, the region consisting of pixels with the intensity equaled to the mean value was taken as nucleus region. Otherwise, the nucleus region was segmented by selecting pixels with intensities that fell in the range of the computed difference and the summation. Morphological closing was employed to fill the small holes in the nucleus region. If more than a single closed region were detected, the region with the largest area was considered as the nucleus. Processing of cervical squamous epithelial cell images is summarized in the flowchart in Fig 1.

### 2.3 Evaluation of Nuclear Membrane Irregularity

**Fig 2:**
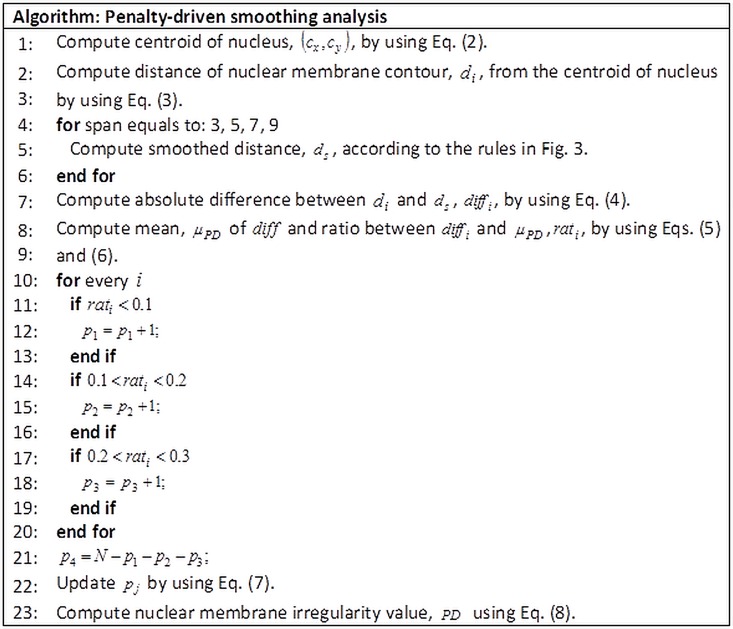
Procedure of the proposed penalty-driven smoothing analysis.

For the evaluation of nuclear membrane irregularity, two techniques were proposed. The first proposed technique, namely, penalty-driven smoothing analysis, smoothed the nuclear membrane contour and employed penalties to the absolute difference between original and smoothed nuclear membrane contours. Averaging filter, a low-pass filter with filter coefficients equaled to the reciprocal of the span, was employed to smooth the nuclear membrane contour. Parameter testing was performed, whereby the span values of 3, 5, 7, and 9 were tested. Absolute difference between the original and smoothed nuclear membrane contour was computed. The ratio between these absolute difference values and the mean value of the absolute difference was obtained. The partitions of nuclear membrane contour that was less than 0.1, fell in the range of 0.1 to 0.2, fell in the range of 0.2 to 0.3, and greater than 0.3 were multiplied with different penalty values (namely, *c*_1_, *c*_2_, *c*_3_, and *c*_4_ respectively). The penalty values, which were generated from linear, quadratic, or cubic function, were introduced to assign different weights so that a more irregular nuclear membrane contour would receive larger penalty. Penalty values employed for the three functions are as listed in Table 1. The procedure of the proposed penalty-driven smoothing analysis is illustrated in Fig 2.

**Table 1:** Penalty values for different functions.

Functions	Penalty Value			
	*c*_1_	*c*_2_	*c*_3_	*c*_4_
Linear	1.0	2.0	3.0	4.0
Quadratic	1.0	4.0	9.0	16.0
Cubic	1.0	8.0	27.0	64.0

For a cervical squamous epithelial cell, the centroid of the nucleus region, as represented by coordinate (*c*_*x*_,*c*_*y*_), was computed as follows:

$$c_{x}=\frac{\sum_{i=1}^{N}x_{i}}{N}$$ 2(a)

$$c_{y}=\frac{\sum_{i=1}^{N}y_{i}}{N}$$ 2(b)

where *x*_*i*_ and *y*_*i*_ are the coordinates of *i*-th point of the nuclear membrane contour and the nuclear membrane contour is build-up of *N* points.

The distance of the nuclear membrane contour, *d*_*i*_, from the centroid of nucleus was computed using

$$d_{i}=\sqrt{{\left(c_{x}-x_{i}\right)}^{2}+{\left(c_{y}-y_{i}\right)}^{2}}\;\text{for}\;i=1,2,\cdots ,N$$ (3)

**Fig 3:**
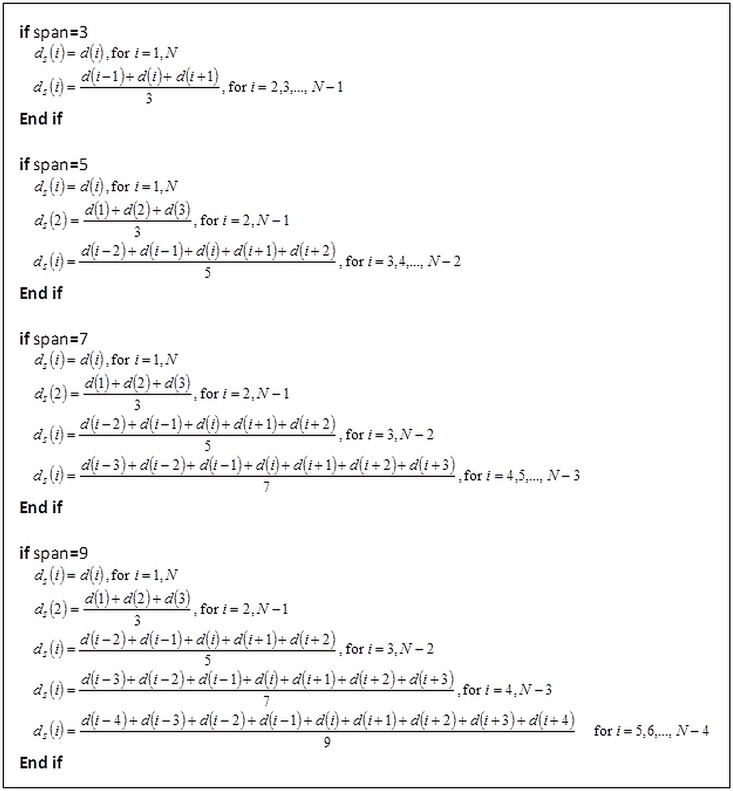
Computation of smoothed distance by using different span values.

The smooth distance with different span values was computed according to Fig 3.

* Note that *d*(1), *d*(2), … *d*(*N*) represent the 1st, 2nd, … *N*-th points of the distance of nuclear membrane contour from the centroid; *d*_*s*_ represents the smoothed distance; and *N* is the total number of points of the nuclear membrane contour.

After the smoothed distance, *d*_*s*_, was obtained using procedure as listed in Fig 3, the absolute difference between the distance of nuclear membrane contour from the centroid and the smoothed distance, *diff*_*i*_, was computed using

$$diff_{i}=\left|d-d_{s}\right|$$ (4)

The mean value of the absolute difference, *μ*_*PD*_, was computed using

$$\mu_{PD}=\frac{\sum_{i=1}^{N}diff_{i}}{N}$$ (5)

The ratio between the absolute difference and the mean value of the absolute difference, *rat*_*i*_, was computed using

$$rat_{i}=\frac{diff_{i}}{\mu_{PD}}$$ (6)

For normalization, dividing the absolute difference with the mean value of the absolute difference normalized the data for comparisons. Therefore, the calculated ratio can be compared directly for the three diagnostic classes. Partition of nuclear membrane difference, *p*_*j*_, that was less than 0.1, fell in the range of 0.1 to 0.2, fell in the range of 0.2 to 0.3, and exceeded 0.3 was computed using

$$p_{j}=p_{j}/N\;\text{for}\;j=1,2,3,4$$ (7)

where *p*_1_, *p*_2_, *p*_3_, and *p*_4_ represent the partition of nuclear membrane difference that is less than 0.1, falls in the range of 0.1 to 0.2, falls in the range of 0.2 to 0.3, and exceeds 0.3, respectively.

Nuclear membrane irregularity, as computed using the penalty driven smoothing analysis, was represented by *PD* and was computed using

$$PD=c_{1}p_{1}+c_{2}p_{2}+c_{3}p_{3}+c_{4}p_{4}$$ (8)

**Fig 4:**
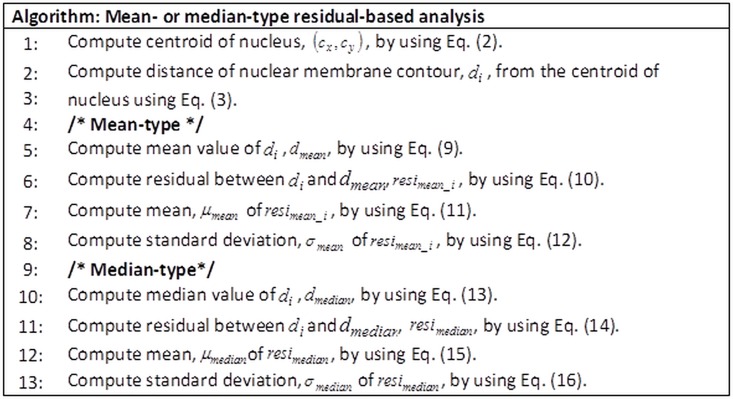
Procedure of the proposed mean- or median-type residual-based analysis.

The second technique, namely, residual-based analysis, evaluated nuclear membrane irregularity based on the residuals of nuclear membrane contour. Distance of the nuclear membrane contour from the centroid of the nucleus region was computed. Two types of residual-based analysis were based on the mean and based on the median. For the mean-type residual-based analysis, residuals of the nuclear membrane contour with the mean of the nuclear membrane contour were computed. In contrast, median-type residual-based analysis computed the residuals of the nuclear membrane contour with the median of the nuclear membrane contour. Then, for both techniques, the mean and standard deviation of the residuals were computed to evaluate the nuclear membrane irregularity. Motivated by the idea of the residuals of a perfect circle will be zero, we anticipate that the mean and standard deviation of the residuals for LSIL and HSIL classes will be greater when compared with the residuals for NILM class. Even though the nuclear membrane contour of NILM class might not be a perfect circle, the variation in shape of LSIL and HSIL classes is suspected to be greater. The procedure for the proposed mean- or median-type residual-based analysis is illustrated in Fig 4.

The mean value for the distance of nuclear membrane contour, *d*_*mean*_, was computed as follows:

$$d_{mean}=\frac{\sum_{i=1}^{N}d_{i}}{N}$$ (9)

Residuals between the mean value for the distance of nuclear membrane contour and the distance of nuclear membrane contour, *resi*_*mean_i*_, were computed using

$$resi_{mean\_i}=\left|d_{i}-d_{mean}\right|\;\text{for}\;i=1,2,\cdots ,N$$ (10)

The mean value of the residuals of the mean, *μ*_*mean*_, was computed using

$$\mu_{mean}=\frac{\sum_{i=1}^{N}resi_{mean\_i}}{N}$$ (11)

Standard deviation of the residuals of the mean, *σ*_*mean*_, was computed using

$$\sigma_{mean}=\sqrt{\frac{1}{N-1}\sum_{i=1}^{N}{\left(resi_{mean\_i}-\mu_{mean}\right)}^{2}}$$ (12)

For median-type residual-based analysis, the median value of the distance of nuclear membrane contour, *d*_*median*_, was computed using

$$d_{median}=\left\{\left(N+1\right)÷2\right\}\text{th value}$$ (13)

where the values for distance of nuclear membrane contour are sorted in ascending order.

Residuals between the median value for the distance of nuclear membrane contour and the distance of nuclear membrane contour, *resi*_*median*_, were computed using

$$resi_{median\_i}=\left|d_{i}-d_{median}\right|\;\text{for}\;i=1,2,\cdots ,N$$ (14)

The mean value of the residuals of the median, *μ*_*median*_, was computed using

$$\mu_{median}=\frac{\sum_{i=1}^{N}resi_{median\_i}}{N}$$ (15)

Standard deviation of the residuals of the median, *σ*_*median*_, was computed using

$$\sigma_{median}=\sqrt{\frac{1}{N-1}\sum_{i=1}^{N}{\left(resi_{median\_i}-\mu_{median}\right)}^{2}}$$ (16)

**Fig 5:**
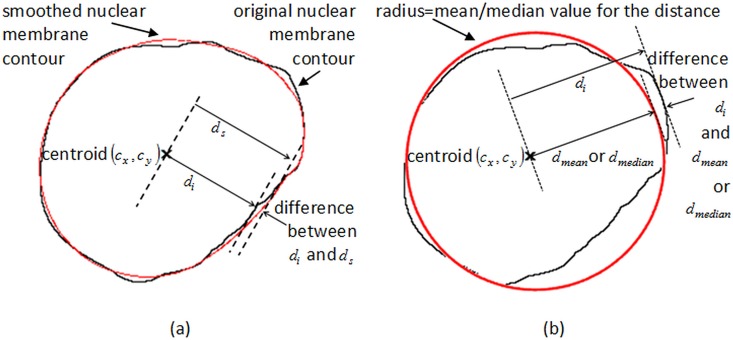
Graphical illustration of (a) penalty-driven smoothing analysis and (b) mean- or median-type residual-based analysis on the same cell.

Graphical illustration for penalty-driven smoothing analysis and residual-based analysis are presented in Fig 5. The original contours of the cells are drawn in black, and the computed contours of the cells are drawn in red. For the penalty-driven smoothing analysis as shown in Fig 5A, the smoothed contour followed closely with the contour of the cell. By contrast, for the residual-based analysis, as shown in Fig 5B, the computed shape is a circle with radius equals the mean or median value of the distance of the nuclear membrane contour.

## 3.0 Results

**Fig 6:**
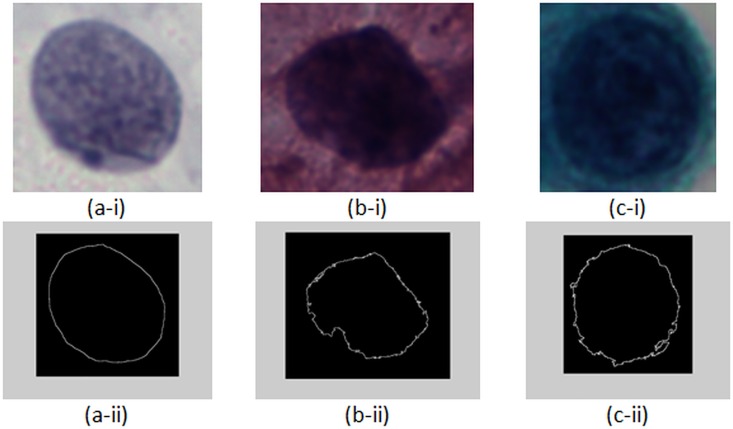
Original nuclear membrane contour of (a) NILM, (b) LSIL, and (c) HSIL as shown in (ii).

**Fig 7:**
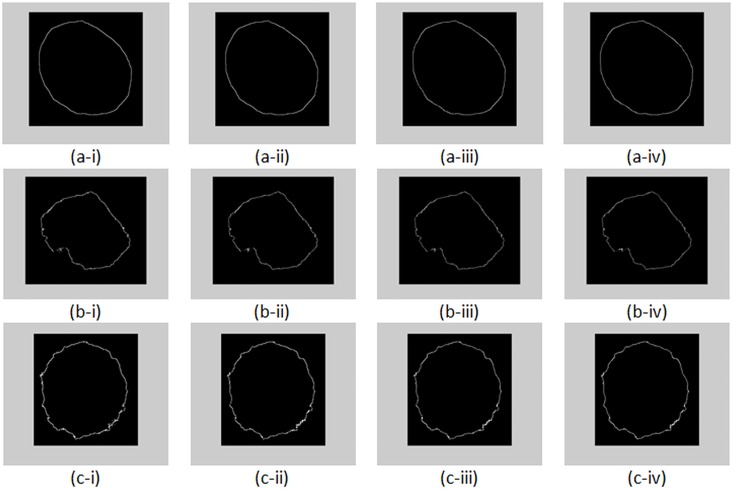
Smoothed nuclear membrane contour of (a) NILM, (b) LSIL, and (c) HSIL by using span = (i) 3, (ii) 5, (iii) 7, and (iv) 9.

Examples of nuclear membrane contours of three cervical squamous epithelial cells from three classes are illustrated in Fig 6. Fig 6 shows that the nucleus of cervical squamous epithelial cell from NILM class presents a more regular contour as compared with nuclei from LSIL and HSIL classes. Nuclear membrane contour smoothed using different span values for cells in Fig 6 are shown in Fig 7. As shown in Fig 7, if the nuclear membrane contour is originally smooth (such as NILM cell), smoothing exerts minimal effect on the contour. As a result, the difference between the original and smoothed contours will be small. For LSIL and HSIL cells, the irregularity is greater compared with the NILM cell. The difference between the original and smoothed contours will be large.

**Fig 8:**
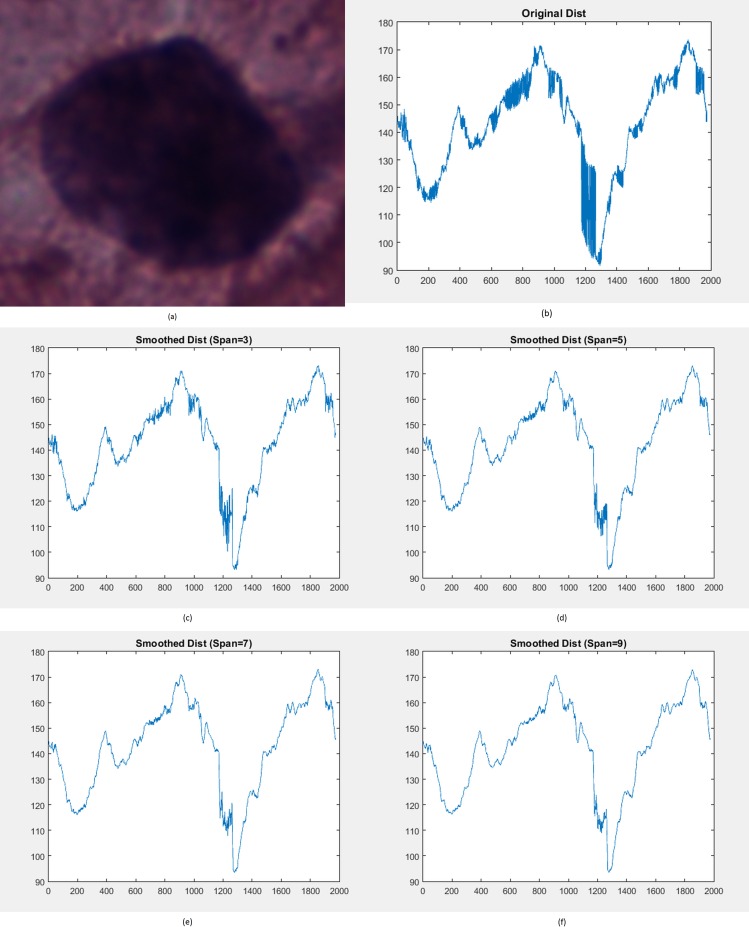
(a) Input nucleus. (b) Original distance of nuclear membrane contour from the centroid of nucleus. Smoothed distance using span = (c) 3, (d) 5, (e) 7, and (f) 9.

As described in the procedure of the proposed penalty-driven smoothing analysis in Fig 2, smoothing is performed on the distance of nuclear membrane contour from the centroid of nucleus. An example of distance smoothing for a LSIL cell is illustrated in Fig 8. Smoothed distance at different span values (that is, span equals 3, 5, 7, and 9) for LSIL cell in Fig 6B–6I are shown in Fig 8C to 8F. The smoothed nuclear membrane contour (illustrated in Fig 7) can be reconstructed based on the smoothed distance.

**Fig 9:**
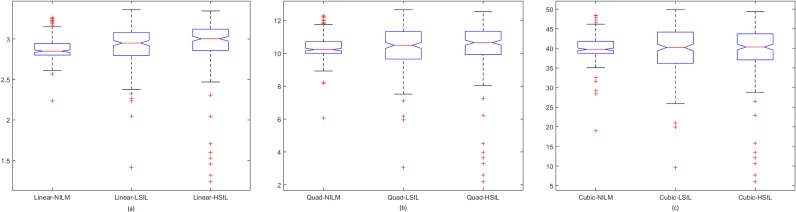
Penalty-driven smoothing analysis with span = 3 and of (a) linear, (b) quadratic, and (c) cubic penalty functions.

**Fig 10:**
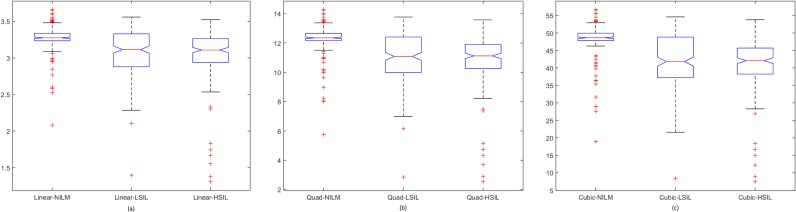
Penalty-driven smoothing analysis with span = 5 and (a) linear, (b) quadratic, and (c) cubic penalty functions.

**Fig 11:**
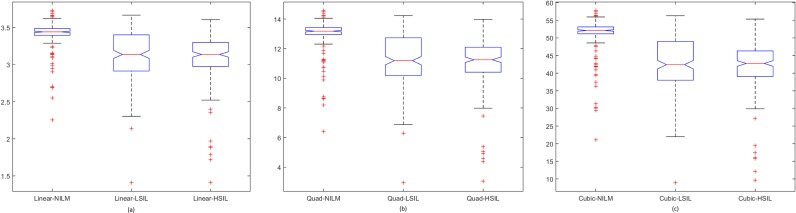
Penalty-driven smoothing analysis with span = 7 and (a) linear, (b) quadratic, and (c) cubic penalty functions.

**Fig 12:**
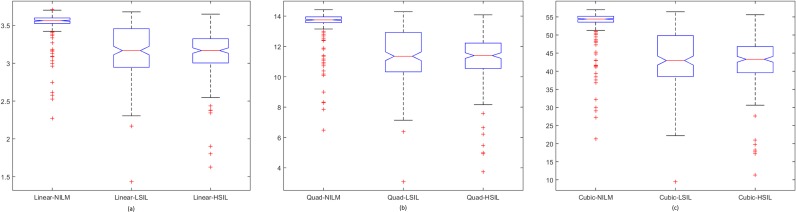
Penalty-driven smoothing analysis with span = 9 and penalty function of (a) linear, (b) quadratic, and (c) cubic penalty functions.

**Fig 13:**
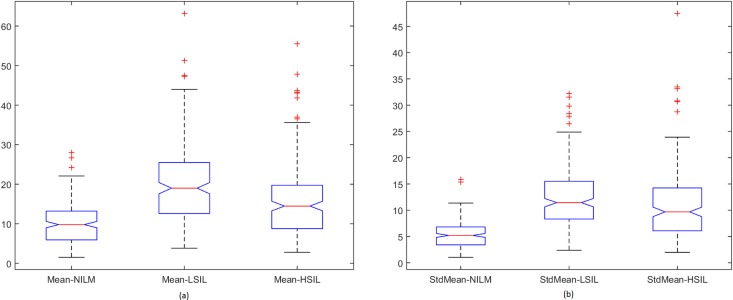
Mean-type residual-based analysis. Boxplot of (a) mean and (b) standard deviation of residuals.

**Fig 14:**
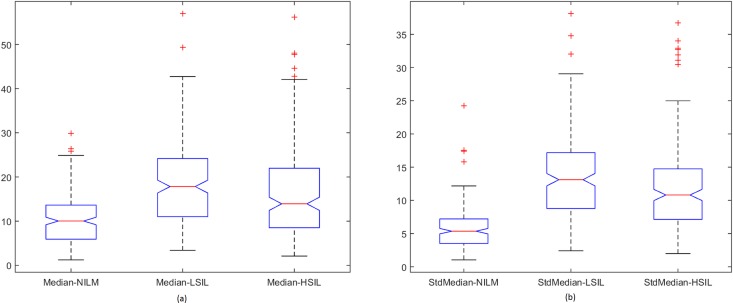
Median-type residual-based analysis. Boxplot of (a) mean and (b) standard deviation of residuals.

**Fig 15:**
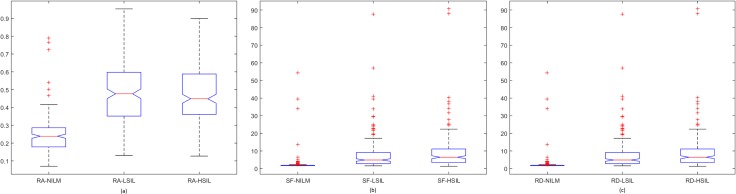
Boxplots of techniques: (a) RA, (b) SF, and (c) RD.

Spread of data for the first proposed technique, the penalty-driven smoothing analysis with four different span values, and three penalty functions are presented in Figs 9–12. For the second technique, the spread of the residuals of the nuclear membrane contour for NILM, LSIL, and HSIL classes from the mean and median values of residuals are presented as boxplots in Figs 13 and 14, respectively. Boxplots of other comparison techniques, namely, RA, SF, and RD, are presented in Fig 15A to 15C, respectively.

For the penalty-driven smoothing analysis, when the span value equaled 3, the median values of the data from NILM class were the minimum among the three classes for all penalty functions. When the span values increased from 5 to 9, both penalty functions and span values demonstrated minimal effect on the spread of data for LSIL and HSIL classes (Figs 10 to 12). Boxplots show similar pattern when employing different penalty functions regardless of span values. NILM class exhibited the largest median value when the span values exceeded 3. Compared with LSIL and HSIL classes, the NILM class showed the smallest range for the data spread. Boxplots in Figs 13 and 14 show that both the mean and standard deviation values of the mean- and median-type residual-based analyses have the smallest range for the NILM class. In addition, residuals of nuclear membrane contour for both techniques were larger for LSIL and HSIL classes, revealing that the shape of the nuclear membranes deviated more in these cells. Minimal overlapping of data occurred for the standard deviation of residuals for both techniques, indicating that NILM could be separated from LSIL and HSIL classes based on the standard deviation of residuals. The boxplots in Figs 13 and 14 appear to be similar as the mean and median of the residuals were not significantly different. Other comparison techniques, such as RA, SF, and RD in Fig 15, demonstrated similar trends to those in the proposed techniques, in which the range of the data was the smallest for the NILM class. Results of the proposed techniques were comparable with those of comparison techniques. Overall, NILM can be separated from LSIL and HSIL classes based on the two proposed techniques or other state-of-art techniques. However, overlapping data can be seen for the LSIL and HSIL classes, resulting in difficulty of separating the two classes based on nuclear membrane irregularity.

To investigate whether these data different significantly, Friedman test was employed for multiple comparisons by using KEEL Software tool [25] with the significance level, *α* equals 0.05 [26]. Friedman test is a non-parametric procedure that investigates the significance of differences between multiple ranks through ranking of the algorithms, where 1st rank is given to the best performing algorithm. Here, a lower value returned for both proposed techniques is considered a superior value. If Friedman test returned a *p*-value less than 0.05, the measurements are significantly different. Friedman test returned statistically significant differences for all comparisons, except for the penalty-driven smoothing analysis with span value equaled 3 using cubic penalty function (*p*-value = 0.5461) as listed in Table 2. Rejection of the null hypothesis leads to post hoc analysis, which aims to obtain pairwise comparisons that yield differences. Holm, Shaffer, and Bergmann procedures were further applied, and the results are summarized in Table 2. These tests were selected based on previous recommendations [26,27], whereby Nemenyi’s test is not recommended owing to its conservative nature, but the use of Shaffer and Holm procedures are strongly recommended. In contrast, Bergmann procedure is the best performing approach despite being computationally expensive. Details of Holm, Shaffer, and Bergmann procedures are presented in S1–S14 Tables. Holm and Shaffer procedures reject all hypotheses if the corresponding *p*-values are smaller than the adjusted *α*’s.

**Table 2:** *P*-values of Friedman test and the summary of classes with significant difference. *P*-value greater than 0.05 is italicized.

No	Technique		*p*-value	Classes with Significant Difference
1	Penalty-driven smoothing analysis			
	3	Linear	5.546E−7	NILM versus HSIL, NILM versus LSIL, LSIL versus HSIL
	3	Quadratic	4.954E−2	NILM versus HSIL
	3	Cubic	*5*.*461E−1*	Nil*
	5	Linear	6.587E−11	NILM versus HSIL, NILM versus LSIL
	5	Quadratic	5.809E−11	NILM versus HSIL, NILM versus LSIL
	5	Cubic	7.650E−11	NILM versus HSIL, NILM versus LSIL
	7	Linear	4.404E−11	NILM versus HSIL, NILM versus LSIL
	7	Quadratic	7.623E−11	NILM versus HSIL, NILM versus LSIL
	7	Cubic	9.359E−11	NILM versus HSIL, NILM versus LSIL
	9	Linear	6.525E−11	NILM versus HSIL, NILM versus LSIL
	9	Quadratic	6.632E−11	NILM versus HSIL, NILM versus LSIL
	9	Cubic	9.692E−11	NILM versus HSIL, NILM versus LSIL
2	Mean-type residual-based analysis			
	Mean		5.278E−11	NILM versus HSIL, NILM versus LSIL, LSIL versus HSIL
	Standard Deviation		1.057E−10	NILM versus HSIL, NILM versus LSIL, LSIL versus HSIL
3	Median-type residual-based analysis			
	Mean		6.264E−11	NILM versus HSIL, NILM versus LSIL, LSIL versus HSIL
	Standard Deviation		1.165E−10	NILM versus HSIL, NILM versus LSIL, LSIL versus HSIL
4	RA		8.823E−11	NILM versus HSIL, NILM versus LSIL
5	SF		1.028E−10	NILM versus HSIL, NILM versus LSIL
6	RD		1.399E−10	NILM versus HSIL, NILM versus LSIL

* Nil: None of the classes present significant difference.

From Table 2, for comparisons of the penalty-driven smoothing analysis using span values equal to 5, 7, and 9, the NILM and LSIL classes and the NILM and HSIL classes presented significant differences. However, LSIL and HSIL classes were not significantly different. A span value of 3 with linear penalty function returned significant difference for all comparisons. By using span value of 3, only one comparison (that is, NILM versus HSIL) returned significant difference when quadratic penalty function was employed, but no significant difference could be found when cubic penalty function was employed. The second proposed technique, i.e., mean- or median-type residual-based analysis, returned significant differences for the comparisons among the three datasets. The three state-of-the-art techniques gave similar results as the majority results from the penalty-driven smoothing analysis, where NILM versus LSIL and NILM versus HSIL class were statistically different, but not for the LSIL versus HSIL class. Based on simulation results, it can be concluded that the diagnostic criteria on nuclear membrane for NILM, LSIL, and HSIL classes could be measured in a quantitative way to reduce vagueness and further increase the reproducibility of judgment.

## 4.0 Discussion

The nuclei of cervical squamous epithelial cells are nearly spherical, which change to oblate spheroidal due to cells flattening during smear preparation. Under microscope, nuclei appear to have near circular profile (that is, round or oval) [18]. Alteration in nuclear shape is either due to changes in the nuclear lamina or by forces from the cytoplasm [28,29]. Abnormality in nuclear shape is correlated to malignancy [11]. In practice, classification of cervical squamous epithelial cells is performed based on the combinations of several diagnostic criteria [30]. This study focused on one of the features, namely, nuclear membrane irregularity, in differentiating the different diagnostic classes of cervical squamous epithelial cells. The diagnostic criteria of the nuclear membrane in one of the reporting standards, the Bethesda system, are listed in Table 3.

**Table 3:** Description for the nuclear membrane in the Bethesda system [30].

Class	Page	Description
NILM	38, 42	Nuclei have smooth contours.
	40, 50	Nuclear contours are regular.
LSIL	138	Contour of nuclear membranes is variable ranging from smooth to very irregular with notches.
HSIL	147	Contour of the nuclear membrane is quite irregular and frequently demonstrates prominent indentations or grooves.

As shown in Table 3, the diagnostic criteria for nuclear membrane are qualitative in nature. Judgment of the descriptive terms, such as smooth and regular, depends on the individual pathologists and is highly subjective depending on his or her skills and experience. Moreover, degree of irregularity is defined through terms such as “quite” and “very”. As a result, discrepancies between individual pathologists are unavoidable, and the diagnostic results may lack of reproducibility. Hence, this study suggests techniques to measure the irregularity in a quantitative way.

**Fig 16:**
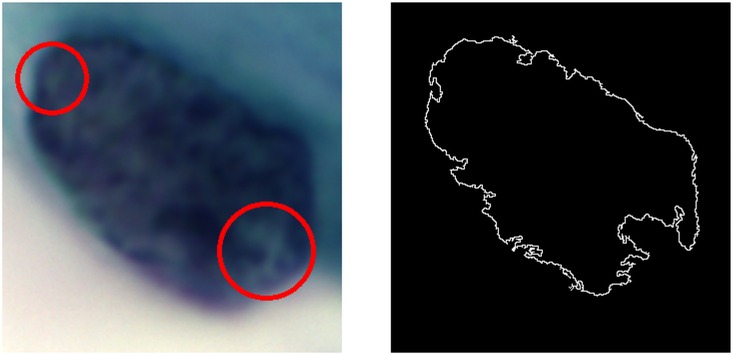
Circles highlight the irregularity in nuclear membrane contour.

Pathologists and cytotechnologists examine and observe the cervical squamous epithelial cells in three-dimensional view by adjusting the focus of the microscope. Abnormality in nuclear membrane for LSIL and HSIL cells, which are characterized as nuclear grooving, nuclear molding, or nuclear convolutions, is aggregated in the two-dimensional image. As such, irregularity in shape yields abrupt change in intensities in the two-dimensional image; a nuclear grooving, which is highlighted and shown in the nuclear membrane contour is illustrated in Fig 16. Capturing images of cervical squamous epithelial cells under microscope can be imagined as capturing an inflatable ball (that is, the NILM cell) and a dented ball (that is, LSIL or HSIL cell). Nuclear membrane irregularity, which appears similar to a dented ball, demonstrates changes in intensity gradient in the two-dimensional image.

The current existing techniques rely on the assumption that the nuclear shape is originally round or symmetrical in shape. By applying the same concept, the proposed mean- or median-type residual-based analysis is designed based on the deviation of nuclear membrane contour from the circle with the radius of mean or median value of the distance of nuclear membrane contour. Simulation results of the proposed mean- or median-type residual-based analysis returned statistical difference among the three classes (i.e., NILM, LSIL, and HSIL). However, there is a concern that the relationship between “round” or “symmetry” and the “regular” as defined by the Bethesda system (Table 3) has yet to be verified. Therefore, the uncertainty in justifying the nuclear shape based on the assumption of “round” or “symmetrical” motivated us to propose the penalty-driven smoothing analysis.

Without making the assumption that the initial shape of the nucleus is round or symmetrical, the proposed penalty-driven smoothing analysis evaluates nuclear membrane irregularity by comparing the original nuclear membrane contour with the smoothed nuclear membrane contour, which is derived from the original nuclear membrane contour. If the nuclear membrane contour is originally smooth, the smoothed profile will be extremely close to the original nuclear membrane contour, resulting in small difference for irregularity measurement. This is a novel approach in evaluating nuclear membrane irregularity as the irregularity is defined based on the smoothness rather than the roundness or symmetrical property.

Different span values were tested in study to investigate the effect of different degrees of smoothing on the evaluation of nuclear membrane irregularity. A suitable span value should yield a smoothed nuclear membrane contour that is loyal to the original nuclear membrane contour and still capable of capturing the point-to-point variance on nuclear membrane contour. A larger span value takes more points into account to smooth the nuclear membrane contour. However, when the span value is extremely large, the smoothed contour would deviate markedly from the original nuclear membrane contour. Although the smoothing effect with different span values are subtle to the naked eye (as shown in Fig 7), results from statistical analysis revealed that span value of 3 was the optimum choice. More specifically, when a span value of 3 was employed with linear penalty function, the proposed penalty driven smoothing analysis showed significant difference among the three diagnostic classes. When span values equaled 5, 7, and 9, NILM class could be separated from LSIL and HSIL classes, but the latter classes were hardly be separated from one another solely based on nuclear membrane irregularity.

For the comparison techniques, RA studies the shape of nucleus via asymmetry, whereas SF studies the degree of deviation from an ideal circle. RA is capable to capture the differences among near-spherical, oval, and irregular shapes of nuclei, whereas SF can hardly distinguish nuclei of oval and irregular shapes [18]. RD could be seen as a variant to the proposed mean- or median-type residual-based analysis, which assumes that the length of the nuclear membrane contour is shorter for a regular nucleus. RD defines nuclear membrane irregularity by comparing the length of the nuclear membrane contour with the length of round (regular) nuclei with the same area. These three comparison techniques evaluate nuclear membrane irregularity from different perspectives and thus serve a suitable comparison to our proposed techniques. Simulation results showed that the proposed techniques in this study yielded comparable outputs with the comparison techniques.

In summary, irregularity can be computed through analysis of the variations in nuclear membrane contour. The proposed techniques in this study are simple yet effective for the evaluation of nuclear membrane irregularity. The issue on the assumption on initial nucleus shape is specifically addressed in this study. The proposed techniques can be employed independently to differentiate the different diagnostic classes of cervical squamous epithelial cells.

## 5.0 Conclusion

Nuclear membrane irregularity is one of the morphological changes related to malignancy. This study proposed two techniques, namely, penalty-driven smoothing analysis and residual-based analysis, to evaluate nuclear membrane irregularity. The former employs different penalty values to a more irregular nuclear membrane contour. Residual-based analysis, which consists of two types of analyses (that is, mean- and median-type), evaluates nuclear membrane irregularity through analyzing the residuals of the nuclear membrane contour from the mean or median value of the nuclear membrane contour. Statistical analyses using Friedman tests returned significant difference with *p*-value less than 0.05 for all comparisons except when the span value was 3 with cubic penalty function. Further tests using Holm, Shaffer, and Bergmann procedures for penalty-driven smoothing analysis returned significant differences for all three classes when the span value of 3 was employed with linear penalty function. Comparisons of NILM versus LSIL and HSIL were significant, but that of LSIL versus HSIL was not significantly different when span values equaled 5, 7, and 9 for all penalty functions. The optimum span value was 3 with the linear penalty function. The residual-based analysis produced significant differences for the three diagnostic classes. The proposed techniques addressed the issue on the assumption on nucleus shape. Findings from this study proved the significance of nuclear membrane irregularity in differentiating the different diagnostic classes of cervical squamous epithelial cells.

## Supporting Information

S1 File Dataset of NILM test images. (RAR)

S2 File Dataset of LSIL test images. (RAR)

S3 File Dataset of HSIL test images. (RAR)

S1 Table Family of hypotheses ordered by *p*-value and adjusting of *α* by Holm and Shaffer procedures, considering an initial *α* = 0.05 for penalty-driven smoothing analysis with span = 3. (DOC)

S2 Table Adjusted *p*-value (APVs) by Holm, Shaffer’s static, and Bergmann-Hommel’s dynamic (Berg) for penalty-driven smoothing analysis with span = 3. (DOC)

S3 Table Family of hypotheses ordered by *p*-value and adjusting of *α* by Holm and Shaffer procedures, considering an initial *α* = 0.05 for penalty-driven smoothing analysis with span = 5. (DOC)

S4 Table Adjusted *p*-value (APVs) by Holm, Shaffer’s static, and Bergmann-Hommel’s dynamic (Berg) for penalty-driven smoothing analysis with span = 5. (DOC)

S5 Table Family of hypotheses ordered by *p*-value and adjusting of *α* by Holm and Shaffer procedures, considering an initial *α* = 0.05 for penalty-driven smoothing analysis with span = 7. (DOC)

S6 Table Adjusted *p*-value (APVs) by Holm, Shaffer’s static, and Bergmann-Hommel’s dynamic (Berg) for penalty-driven smoothing analysis with span = 7. (DOC)

S7 Table Family of hypotheses ordered by *p*-value and adjusting of *α* by Holm and Shaffer procedures, considering an initial *α* = 0.05 for penalty-driven smoothing analysis with span = 9. (DOC)

S8 Table Adjusted *p*-value (APVs) by Holm, Shaffer’s static, and Bergmann-Hommel’s dynamic (Berg) for penalty-driven smoothing analysis with span = 9. (DOC)

S9 Table Family of hypotheses ordered by *p*-value and adjusting of *α* by Holm and Shaffer procedures, considering an initial *α* = 0.05 for mean-type residual-based analysis. (DOC)

S10 Table Adjusted *p*-value (APVs) by Holm, Shaffer’s static, and Bergmann-Hommel’s dynamic (Berg) for mean-type residual-based analysis. (DOC)

S11 Table Family of hypotheses ordered by *p*-value and adjusting of *α* by Holm and Shaffer procedures, considering an initial *α* = 0.05 for median-type residual-based analysis. (DOC)

S12 Table Adjusted *p*-value (APVs) by Holm, Shaffer’s static, and Bergmann-Hommel’s dynamic (Berg) for median-type residual-based analysis. (DOC)

S13 Table Family of hypotheses ordered by *p*-value and adjusting of *α* by Holm and Shaffer procedures, considering an initial *α* = 0.05 for RA, SD and RD techniques. (DOC)

S14 Table Adjusted *p*-value (APVs) by Holm, Shaffer’s static, and Bergmann-Hommel’s dynamic (Berg) for RA, SD and RD techniques. (DOC)
