# Supplementary material for: Evaluating Nuclear Membrane Irregularity for the Classification of Cervical Squamous Epithelial Cells
Source: PLoS One. 2016 Oct 14;11(10):e0164389. doi: 10.1371/journal.pone.0164389 (PMC5065206; doi:10.1371/journal.pone.0164389)
Supplement: S3 Table — (DOC) [file pone.0164389.s006.doc]

**Table S3. Family of hypotheses ordered by *p*-value and adjusting of *α* by Holm and Shaffer procedures, considering an initial *α* = 0.05 for penalty-driven smoothing analysis with span = 5.**

| i | Hypothesis | z | p | | α_Holm_ | | α_Shaffer_ | |  |
| --- | --- | --- | --- | --- | --- | --- | --- | --- | --- |
| Linear | |  | |  | |  | |  | |
| 1 | NILM vs. HSIL | 7.00 | 0 | | 0.016667 | | 0.016667 | |  |
| 2 | NILM vs. LSIL | 6.05 | 0 | | 0.025000 | | 0.050000 | |  |
| 3 | LSIL vs. HSIL | 0.95 | 0.342112 | | 0.050000 | | 0.050000 | |  |
| Quadratic | |  | |  | |  | |  | |
| 1 | NILM vs. HSIL | 7.80 | 0 | | 0.016667 | | 0.016667 | |  |
| 2 | NILM vs. LSIL | 7.05 | 0 | | 0.025000 | | 0.050000 | |  |
| 3 | LSIL vs. HSIL | 0.75 | 0.453255 | | 0.050000 | | 0.050000 | |  |
| Cubic | |  | |  | |  | |  | |
| 1 | NILM vs. HSIL | 8.40 | 0 | | 0.016667 | | 0.016667 | |  |
| 2 | NILM vs. LSIL | 7.65 | 0 | | 0.025000 | | 0.050000 | |  |
| 3 | LSIL vs. HSIL | 0.75 | 0.453255 | | 0.050000 | | 0.050000 | |  |
