# Supplementary material for: Evaluating Nuclear Membrane Irregularity for the Classification of Cervical Squamous Epithelial Cells
Source: PLoS One. 2016 Oct 14;11(10):e0164389. doi: 10.1371/journal.pone.0164389 (PMC5065206; doi:10.1371/journal.pone.0164389)
Supplement: S10 Table — (DOC) [file pone.0164389.s013.doc]

**Table S10. Adjusted *p*-value (APVs) by Holm, Shaffer’s static, and Bergmann-Hommel’s dynamic (Berg) for mean-typed residual-based analysis.**

| i | Hypothesis | Unadjusted p_i_ | | APV_Holm_ | | APV_Shaffer_ | | APV_Berg_ | |
| --- | --- | --- | --- | --- | --- | --- | --- | --- | --- |
| Mean of Residuals | |  |  | |  | |  | |  |
| 1 | NILM vs. LSIL | 0 | | 0 | | 0 | | 0 | |
| 2 | NILM vs. HSIL | 0 | | 0 | | 0 | | 0 | |
| 3 | LSIL vs. HSIL | 0.000001 | | 0.000001 | | 0.000001 | | 0.000001 | |
| Standard Deviation of Residuals | |  |  | |  | |  | |  |
| 1 | NILM vs. LSIL | 0 | | 0 | | 0 | | 0 | |
| 2 | NILM vs. HSIL | 0 | | 0 | | 0 | | 0 | |
| 3 | LSIL vs. HSIL | 0.001935 | | 0.001935 | | 0.001935 | | 0.001935 | |
