# Supplementary material for: Evaluating Nuclear Membrane Irregularity for the Classification of Cervical Squamous Epithelial Cells
Source: PLoS One. 2016 Oct 14;11(10):e0164389. doi: 10.1371/journal.pone.0164389 (PMC5065206; doi:10.1371/journal.pone.0164389)
Supplement: S11 Table — (DOC) [file pone.0164389.s014.doc]

**Table S11. Family of hypotheses ordered by *p*-value and adjusting of *α* by Holm and Shaffer procedures, considering an initial *α* = 0.05 for median-typed residual-based analysis.**

| i | Hypothesis | z | p | | α_Holm_ | | α_Shaffer_ | |  |
| --- | --- | --- | --- | --- | --- | --- | --- | --- | --- |
| Mean of Residuals | |  | |  | |  | |  | |
| 1 | NILM vs. LSIL | 8.70 | 0 | | 0.016667 | | 0.016667 | |  |
| 2 | NILM vs. HSIL | 5.70 | 0 | | 0.025000 | | 0.050000 | |  |
| 3 | LSIL vs. HSIL | 3.00 | 0.002700 | | 0.050000 | | 0.050000 | |  |
| Standard Deviation of Residuals | |  | |  | |  | |  | |
| 1 | NILM vs. LSIL | 11.80 | 0 | | 0.016667 | | 0.016667 | |  |
| 2 | NILM vs. HSIL | 9.65 | 0 | | 0.025000 | | 0.050000 | |  |
| 3 | LSIL vs. HSIL | 2.15 | 0.031555 | | 0.050000 | | 0.050000 | |  |
