# Supplementary material for: Evaluating Nuclear Membrane Irregularity for the Classification of Cervical Squamous Epithelial Cells
Source: PLoS One. 2016 Oct 14;11(10):e0164389. doi: 10.1371/journal.pone.0164389 (PMC5065206; doi:10.1371/journal.pone.0164389)
Supplement: S13 Table — (DOC) [file pone.0164389.s016.doc]

**Table S13. Family of hypotheses ordered by *p*-value and adjusting of *α* by Holm and Shaffer procedures, considering an initial *α* = 0.05 for RA, SD and RD techniques.**

| i | Hypothesis | z | p | | α_Holm_ | | α_Shaffer_ | |  |
| --- | --- | --- | --- | --- | --- | --- | --- | --- | --- |
| RA | |  | |  | |  | |  | |
| 1 | NILM vs HSIL | 11.75 | 0 | | 0.016667 | | 0.016667 | |  |
| 2 | NILM vs LSIL | 11.62 | 0 | | 0.025000 | | 0.050000 | |  |
| 3 | LSIL vs HSIL | 0.10 | 0.920344 | | 0.050000 | | 0.050000 | |  |
| SF | |  | |  | |  | |  | |
| 1 | NILM vs. HSIL | 13.45 | 0 | | 0.033333 | | 0.033333 | |  |
| 2 | NILM vs. LSIL | 12.20 | 0 | | 0.050000 | | 0.100000 | |  |
| 3 | LSIL vs. HSIL | 1.25 | 0.211300 | | 0.100000 | | 0.100000 | |  |
| RD | |  | |  | |  | |  | |
| 1 | NILM vs. HSIL | 13.50 | 0 | | 0.016667 | | 0.016667 | |  |
| 2 | NILM vs. LSIL | 12.30 | 0 | | 0.025000 | | 0.050000 | |  |
| 3 | LSIL vs. HSIL | 1.20 | 0.230139 | | 0.050000 | | 0.050000 | |  |
