# Supplementary material for: Evaluating Nuclear Membrane Irregularity for the Classification of Cervical Squamous Epithelial Cells
Source: PLoS One. 2016 Oct 14;11(10):e0164389. doi: 10.1371/journal.pone.0164389 (PMC5065206; doi:10.1371/journal.pone.0164389)
Supplement: S14 Table — (DOC) [file pone.0164389.s017.doc]

**Table S14. Adjusted *p***-value (APVs) by Holm, Shaffer’s static, and Bergmann-Hommel’s dynamic (Berg) for RA, SD and RD techniques.

| i | Hypothesis | Unadjusted p_i_ | | APV_Holm_ | | APV_Shaffer_ | | APV_Berg_ | |
| --- | --- | --- | --- | --- | --- | --- | --- | --- | --- |
| RA | |  |  | |  | |  | |  |
| 1 | NILM vs. HSIL | 0 | | 0 | | 0 | | 0 | |
| 2 | NILM vs. LSIL | 0 | | 0 | | 0 | | 0 | |
| 3 | LSIL vs. HSIL | 0.920344 | | 0.920344 | | 0.920344 | | 0.920344 | |
| SF | |  |  | |  | |  | |  |
| 1 | NILM vs. HSIL | 0 | | 0 | | 0 | | 0 | |
| 2 | NILM vs. LSIL | 0 | | 0 | | 0 | | 0 | |
| 3 | LSIL vs. HSIL | 0.211300 | | 0.211300 | | 0.211300 | | 0.211300 | |
| RD | |  |  | |  | |  | |  |
| 1 | NILM vs. HSIL | 0 | | 0 | | 0 | | 0 | |
| 2 | NILM vs. LSIL | 0 | | 0 | | 0 | | 0 | |
| 3 | LSIL vs. HSIL | 0.230139 | | 0.230139 | | 0.230139 | | 0.230139 | |
